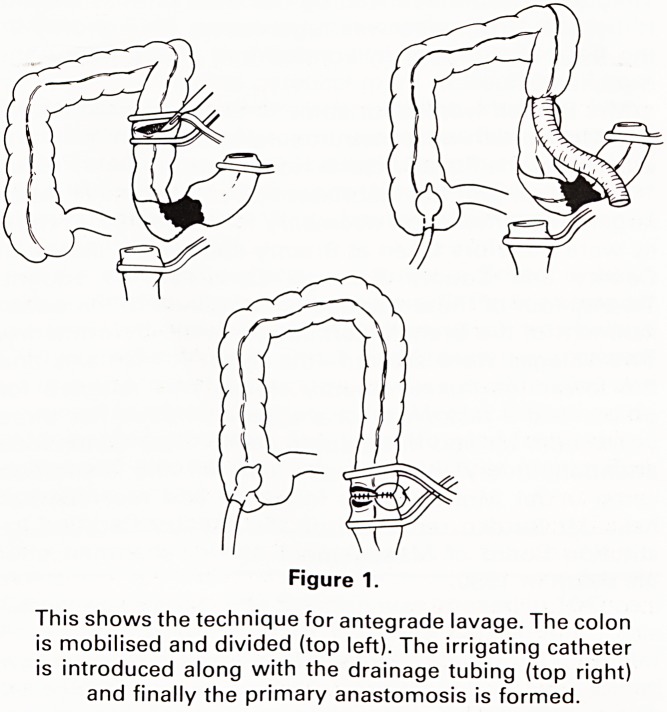# Antegrade Colonic Lavage in Acute Colonic Obstruction

**Published:** 1986-06

**Authors:** Michael E. Foster, Colin D. Johnson

**Affiliations:** Department of Surgery, Southmead Hospital, Bristol; Department of Surgery, Southmead Hospital, Bristol

**Keywords:** Colon, acute obstruction, bowel preparation, anastomosis

## Abstract

Conventional management of acute left sided colonic obstruction employs some form of proximal colostomy. Intraoperative antegrade colonic irrigation relieves proximal faecal loading and may permit safer primary resection and anastomosis. The results of a pilot study are presented, and are shown to be favourable.


### Antegrade Colonic Lavage in the Management of Acute Colonic Obstruction

**Michael E. Foster, FRCS and Colin D. Johnson, FRCS.**
Department of Surgery, Southmead Hospital, Bristol

**ABSTRACT**

Conventional management of acute left sided colonic obstruction employs some form of proximal colostomy. Intraoperative antegrade colonic irrigation relieves proximal faecal loading and may permit safer primary resection and anastomosis. The results of a pilot study are presented, and are shown to be favourable.

**KEY WORDS**

Colon, acute obstruction, bowel preparation, anastomosis.

### INTRODUCTION

Conventional management of patients presenting with acute obstructing colonic lesions using a preliminary transverse colostomy, followed by resection and subsequent colostomy closure, evolved because of the danger associated with primary anastomosis in the presence of distended bowel (1). The gross proximal faecal loading, bacterial overgrowth and perhaps decreased blood supply to distended colon have all been implicated as factors contributing to subsequent anastomotic failure (2).

Staged operations however, may lead to an increased mortality in the elderly and many patients are considered unfit for second or third procedures and are left with permanent colostomies (3, 4). In a recent survey in Bristol 16% of colostomy closures resulted in faecal leakage emphasising the potential problems associated with staged procedures (5).

In order to avoid these problems Dudley, Radcliffe and McGeehan modified a technique of on-table colonic lavage originally described by Muir in 1968 (6, 7). This technique facilitated primary anastomosis in acute left sided colonic obstruction. We have recently employed this technique in a pilot study of primary anastomosis for acute large bowel obstruction in Southmead Hospital, Bristol.

### PATIENTS AND METHODS

Over a 9 month period between October 1984 and June 1985, ten patients presenting with acute left colonic obstruction necessitating urgent laparotomy were managed by antegrade colonic lavage.

After initial resuscitation a midline laparotomy was performed. The obstructing lesion was identified, the left colon and sigmoid mobilised and divided below the site of obstruction. Through a caecostomy or appendicostomy a 26 F Foley catheter was introduced and anchored via a purse-string suture. Through a transverse colotomy incision, immediately proximal to the obstruction, a

Address for correspondence: M. E. Foster, Department of Surgery, Cardiff Royal Infirmary, Cardiff.

**Figure 1.**

This shows the technique for antegrade lavage. The colon is mobilised and divided (top left). The irrigating catheter is introduced along with the drainage tubing (top right) and finally the primary anastomosis is formed.

length of sterile anaesthetic scavenger tubing was introduced into the colon and allowed to lie over the side of the abdominal wound and into a bucket or plastic bag on the theatre floor. Normal saline was introduced into the colon through the Foley catheter in order to irrigate the proximal obstructed bowel and empty this part of the colon of faecal loading (Figure 1). Approximately 3 litres was required before the faecal effluent was clear. The scavenger tubing was removed, the colon, including obstructing lesion, resected and a primary end to end colo-colic anastomosis performed. In four cases the Foley catheter was retained post-operatively to act as a temporary caecostomy; in the remaining patients the catheter was removed after the irrigation was completed.

### RESULTS

There were six females and four males in this pilot study. The age range was 52–82 years with a median of 76 years. Table 1 shows the aetiology of the acute obstruction.

The length of the operation was assessed from the anaesthetic time noted and varied from 80-190 minutes with a median of 130 minutes. The length of stay ranged from 10–30 days with a median stay of 18 days.

No patient suffered from a clinical apparent anastomotic leakage. Two patients suffered minor post-operative wound infections. Although no firm data was available, it was the impression of the operating surgeons that the length of postoperative ileus was protracted compared to that seen following elective colonic resection. In one patient the first bowel action did not occur until the eleventh post-operative day.

**Table 1**

| *Aetiology of acute colonic obstruction* | *Numbers of patients* |
|---|---|
| Obstructing carcinoma (sigmoid or rectum) (1A, 1B, 2C) | 4 |
| Diverticular disease | 4 |
| Sigmoid volvulus | 2 |
| Total | 10 Total |

### DISCUSSION

Although resection and primary anastomosis for acute colonic obstruction is generally considered hazardous, several workers have reported favourable results. The overall incidence of anastomotic leakage varies between 0–12% for elective colonic resection, but emergency resection is associated with an increased risk of anastomatic failure (2,8). However, using assiduous emptying of the large bowel, though not antegrade lavage, White and Macfie recently reported a 10% anastomotic leak rate following primary anastomosis in acutely obstructed left colon (9).

The detrimental effects of faecal loading on anastomotic healing are well recognised and the need for mechanical bowel preparations in elective colonic surgery is widely accepted. In this present small series we have been favourably impressed by the efficacy of antegrade lavage for on-table bowel preparation. Several minor problems have become apparent. Sterilisation of the scavenger tubing can only be achieved by soaking in Cidex which should be washed off before use. The risk of faecal spillage and subsequent wound contamination can be reduced by adopting a closed system of drainage, emptying the scavenger tubing into a plastic bag – this also relieves the malodorous aroma!

The technique of on-table lavage allows a one-staged procedure to be performed, and this avoids the surgical and social complications of colostomy which haunt many elderly patients. Although the duration of operating time is often longer than that of a comparable elective procedure, this must be balanced against the combined duration of a two or three staged operation. The length of stay in this series is certainly shorter than that required for conventional staged management.

The philosophy of proximal colostomy with or without immediate resection for acute left sided obstructed must now be questioned. Both antegrade lavage or extended right hemicolectomy with ileo-sigmoid anastomosis are effective one staged procedures which avoid all the problems of colostomy (6,10). In the present series temporary caecostomy was employed in 40% of patients but this appears to be unnecessary, indeed a persistent faecal discharge has complicated one case, and it's only advantage is for the assessment of anastomotic integrity by post-operative antegrade contrast enema.

Perhaps the time is ripe for a more widespread clinical assessment of intra-operative lavage or sub-total colectomy in the management of acute left side colonic obstruction.

### ACKNOWLEDGEMENT

We thank the Consultant Surgeons of Southmead Hospital for allowing us to report these cases.

### REFERENCES

1. GOLIGHER, J. C. (1975). Surgery of the Anus, Colon and Rectum. 3rd ed. Eastbourne, Balliere Tindall.
2. FOSTER, M. E. & LEAPER, D. J. (1985) The Alimentary Tract. In: Wound healing for Surgeons. (eds.) T. E. Bucknall & H. Ellis, Eastbourne, Balliere Tindal.
3. IRVIN, T. T. & GREANEY, M. G. (1977). The treatment of colonic cancer presenting with acute intestinal obstruction. *Br.J.Surg.* **64**, pp. 741–744.
4. FIELDING, L. P., STEWART-BROWN, S. & BLESOVSKY, L. (1979). Large bowel obstruction caused by cancer: a prospective study. *Br.Med.J.* **2**, pp. 515–517.
5. FOSTER, M. E., WILLIAMSON, R. C. N. & LEAPER, D. J. (1985). Changing patterns in colostomy closure – the Bristol experience, 1975–1982. *Br.J.Surg.* **72**, pp. 142–146.
6. DUDLEY, H. A., RADCLIFFE, A. G. & McGEEHAN, D. Intra-operative irrigation of the colon to permit primary anastomosis.
7. MUIR, E. G. (1968). Safety in colonic resection. *Proc.-Roy.Soc.Med.* **61**, 401.
8. SCHROCK, R. T., DERENEY, C. W. & DUMPTY, J. E. (1973). Factors contributing to leakage of colonic anastomosis. *Ann.Surg.* **177**, pp. 513–518.
9. WHITE, C. M. & MACFIE, J. (1985). Immediate colectomy and primary anastomosis for acute obstruction due to carcinoma of the left colon and rectum. *Dis.Colon Rectum.* **28**, pp. 155–157.
10. HUGHES, E. S. R., McDERMOTT, F. T., POLGLASE, A. L. & NOTTLE P. (1985). Total and sub-total colectomy for colonic obstruction. *Dis.Colon Rectum.* **28**, pp. 162–163.

## Figures and Tables

**Figure 1. f1:**